# Relative Displacement Method for Track-Structure Interaction

**DOI:** 10.1155/2014/397515

**Published:** 2014-01-22

**Authors:** Frank Schanack, Óscar Ramón Ramos, Juan Patricio Reyes, Marcos J. Pantaleón

**Affiliations:** ^1^Institute of Civil Engineering, Universidad Austral de Chile, General Lagos, 5111187 Valdivia, Chile; ^2^Department of Structural and Mechanical Engineering, University of Cantabria, Avenida Los Castros s/n, 39005 Santander, Spain; ^3^APIA XXI S.A., PCTCAN, Avenida Albert Einstein 6, 39011 Santander, Spain

## Abstract

The track-structure interaction effects are usually analysed with conventional FEM programs, where it is difficult to implement the complex track-structure connection behaviour, which is nonlinear, elastic-plastic and depends on the vertical load. The authors developed an alternative analysis method, which they call the relative displacement method. It is based on the calculation of deformation states in single DOF element models that satisfy the boundary conditions. For its solution, an iterative optimisation algorithm is used. This method can be implemented in any programming language or analysis software. A comparison with ABAQUS calculations shows a very good result correlation and compliance with the standard's specifications.

## 1. Introduction

Since the 1980s, the track-structure interaction in railway bridges has been the subject of research, especially since the beginning of the high speed railway traffic in Europe [1–4]. These studies refer to the stresses and deformations in the rail-deck system, which may reach unsafe values and can affect the serviceability of the track. The rail stress may even be high enough to cause its rupture [5]. Generally, such effects occur in continuously welded rails, which are currently being used in high speed railway tracks because of their superior maintainability and passenger comfort [6].

Usually, the combined response of track and structure is analysed by standard finite element analysis software [5, 7–10]. The major challenge of this type of analysis is the implementation of the connector element between rail and bridge deck, which has a nonlinear mechanical behaviour and is elastic-plastic with irreversible deformations and moreover depends on the value of the vertical load. Much of the commercial finite element software is not prepared for these tasks, especially the last one.

The authors propose a different method for the analysis of the effects of the track-structure interaction. It is based on the calculation of deformation states in single DOF finite element models that satisfy the boundary conditions of the track and structure. For its solution, an iterative optimisation algorithm should be used instead of the solution of the system of equations by means of a stiffness matrix. This method can be implemented in any programming language or analysis software, such as FORTRAN, MATLAB, MathCAD, or even EXCEL. Furthermore, any mechanical behaviour of the connector element can be incorporated easily. The authors call it the relative displacement method.

In this work, the concept of the new formulation is derived, and the results of a comparison with the conventional method for the loads creep, shrinkage, and temperature variation are presented.

## 2. The Track-Structure Interaction Phenomenon

### 2.1. Structural Behaviour

The track-structure interaction or the combined response of the structure and track describes the effects of the structural collaboration of the rails and the deck in bridges by means of their connection elements. In the beginning, the analysis of the rails and bridge deck was conducted separately. However, this type of analysis is not appropriate when the rails are continuously welded on top of the structure because then the track-structure interaction shows nonnegligible effects [6, 11].

**Figure 1:**
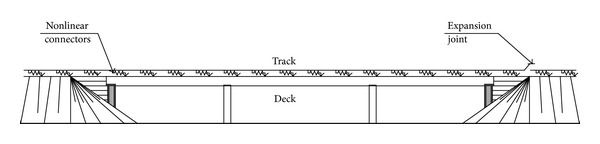
Usual analysis model of the track-structure interaction.

The track-structure interaction analysis is based on the model shown in Figure 1. The track and the deck are modelled by beam elements in their respective centres of gravity. Both parts are connected by the ballast, which transfers forces between them. It is modelled by longitudinal connectors with certain nonlinear mechanical behaviour. Usually, this analysis is conducted with conventional finite element software.

**Figure 2:**
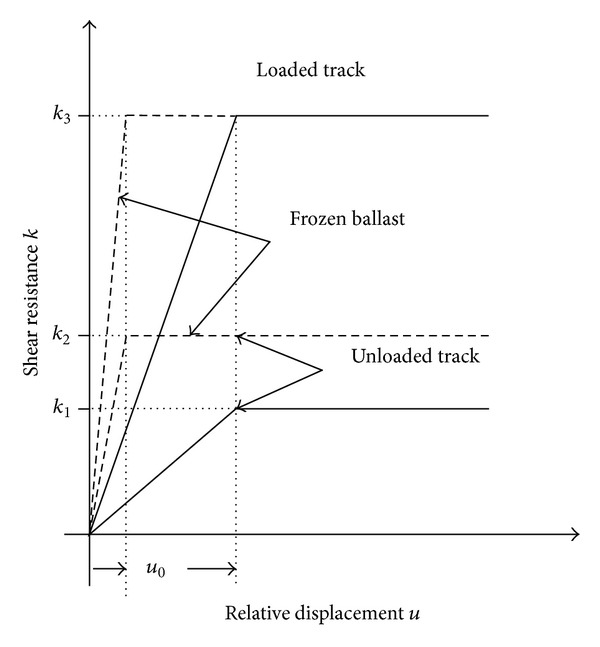
Load-displacement behaviour of ballasted tracks [12].

In the case of ballasted tracks, the structural collaboration of rail and structure is not rigid. It is generally accepted that the load-displacement behaviour of the ballast can be idealised by the bilinear law shown in Figure 2, similar to frictional behaviour [9–14].

The longitudinal shear resistance of the ballast, *k*, is proportional to the displacement of the rail relative to the top of the supporting deck, *u*, until a relative displacement of *u*_0_ is reached, which corresponds to an elastic limit. At this point, the ballast cannot resist any further load, and a sliding phenomenon occurs, while the resistance force is constant (plastic shear resistance). When the direction of the displacement changes, the ballast behaviour becomes elastic again, but the relative displacement from sliding is not recovered. The elastic limit is different for frozen and unfrozen ballasts.

Analogously to frictional behaviour, the plastic shear resistance of the ballast is higher when an additional vertical load is applied, which is the case when the live load is applied to the track (Figure 2). Hence, the analysis must take into account, for example, that the connector elements that are in the sliding state before applying the live load will return to elastic behaviour, while their relative displacement and their connector force remain unchanged. The implementation of such a connector in the analysis of the interaction phenomenon with the finite element method causes certain complications, such as the activation and deactivation of elements in function of the presence of load, and cannot be realised in many engineering FEM programs.

### 2.2. Actions on the Track-Structure System

It is necessary to take into account all actions that may cause longitudinal forces or displacements both in the track and the structure. These actions may be of very different nature, as, for example, creep and shrinkage, temperature variation, stress from vertical loads, or traction and braking forces. Any of these actions can cause a force transfer between the rail and deck via the rail fasteners and the ballast [12].

The present work focuses on the actions that cause the greatest relative displacements between the track and the structure, that is, creep and shrinkage and the variation of the temperature of the deck and rails. Nevertheless, the proposed method can be used to calculate the effects of any of the actions mentioned above.

#### 2.2.1. Creep and Shrinkage

In concrete bridges, part of the creep and shrinkage phenomenon occurs after the installation of the track. This part has to be taken into account for the track-structure interaction analysis. It produces a deck shortening such that every point of the deck moves towards the fixed bearing of the bridge, which usually is located at one abutment. Consequently, the creep and shrinkage strains have a defined direction.

The result is a permanent stress state of the rail-structure connection, which will certainly disappear in time due to the dynamic actions of the passing trains. To take into account the most unfavourable condition, it is prudent to analyse the two possibilities, the presence and the absence of the imposed stress state due to creep and shrinkage.

#### 2.2.2. Variation of the Rail and the Deck Temperature

In general, the value of the constant temperature variation depends on the bridge type and the climatic zone of its placement. For the deck temperature variation, the overall range of the uniform temperature component according to the Eurocode [15] is considered. In the National Annexes, alternative values may be specified. For example, in the Spanish railway bridge design code IAPF-07, the maximum deck temperature variation is ±35 K, while the maximum rail temperature variation is ±50 K. The maximum temperature difference between both elements is ±20 K [13].

### 2.3. Required Verifications

The combined response of track and structure can have unfavourable effects on the bridge structure that have to be considered for its dimensioning. Additionally, there are unfavourable effects on the track-ballast system that can affect the security and the functionality of the bridge. According to Eurocode 1, the main verifications to be conducted are the following [12].

- The additional rail stresses due to the combined response of the structure and track to variable actions should be limited to 72 N/mm^2^ in compression and 92 N/mm^2^ in tension. In continuously welded rails, the stress increment is calculated with respect to the rail stress in the rail at a sufficiently large distance from the bridge. The given values correspond to the commonly used UIC 60 rail with a tensile strength of at least 900 N/mm².
- The absolute deck displacement at both ends of the bridge due to traction and braking shall not exceed 5 mm. If there are rail expansion joints at both ends of the bridge, this displacement shall not exceed 30 mm.
- Additionally, in some National Annexes, a limit of 4 mm is specified for the relative longitudinal displacement of deck and rail due to traction and braking [13, 14].

## 3. Alternative Analysis Method

### 3.1. Concept

During the analysis of 15 high speed railway bridges for Spanish AVE tracks, the authors recognised that the implementation of the mechanical connector behaviour, as described before, is rather complicated, even in very advanced FEM software, such as ABAQUS. In particular the stiffness change due to vertical loading requires additional programming effort.

To reduce the complexity of the problem, the authors derived an alternative analysis method that is based on finite elements with a single degree of freedom, that is, the displacement in longitudinal direction. Both the track and the bridge deck are modelled with these elements. The connection between the track and structure is taken into account as forces applied to track and structure nodes. The force value is obtained from the actual relative displacement and the relative displacement history, according to Figure 2. In the same way, any longitudinal load and the restoring forces from piers and bearings are taken into account at the respective rail and deck nodes.

Under given longitudinal loads from traction, braking, or seismic actions and imposed longitudinal strains from creep and shrinkage or temperature actions, an infinite number of deformation states of such a model can be found. However, only one of these deformation states will satisfy the boundary conditions of the analysis problem. This special equilibrium state can easily be determined by any iterative optimisation algorithm, without the need to solve a system of equations by means of a stiffness matrix. The authors first programmed this analysis method as an EXCEL worksheet and then utilised a FORTRAN program due to the higher precision and the faster mathematical operators.

The output of this method includes all displacements, strains and forces of the track, the structure, and their connection.

**Figure 3:**
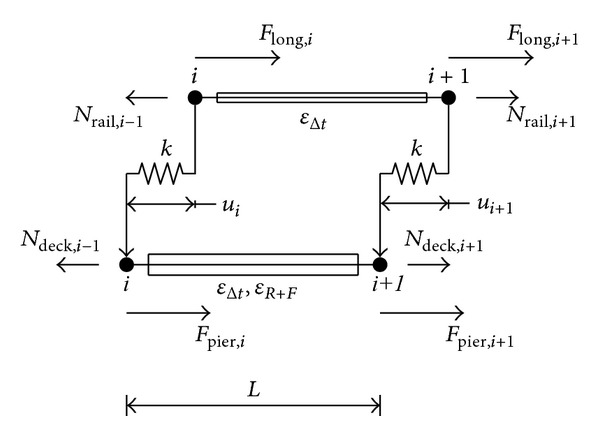
Illustration of the alternative calculation model.

Figure 3 shows a schematic representation of the alternative analysis model. There are two parallel elements, one for the track and one for the deck, with their respective elongation stiffness. The element length, *L*, is determined in the same manner as in usual FEM bridge models. Good results are obtained for a length of 1 m. The required mathematical precision of this method is not altered by the element length.

The ballast is represented by a connector element that can be defined with any mechanical behaviour, in this case, nonlinear and elastic-plastic, as a function of the vertical load. The connector force on the left, acting between the nodes *i* of the rail and of the deck, depends on their relative displacement, which is given as a result of the previous analysis of the adjacent left-hand element. The relative displacement of the nodes *i* + 1 is then obtained from the determination of the total element strain of the track and of the deck due to stress and imposed strain, as shown in

(1) $$\begin{array}{c} u_{i+1}=u_{i}+\left(\varepsilon_{\text{rail},i}-\varepsilon_{\text{deck},i}\right)\cdot L,\\ \varepsilon_{\text{rail},i}^{\text{total}}=\frac{\sigma_{\text{rail},i}}{E_{\text{rail}}}+\varepsilon_{\text{rail},i},\\ \varepsilon_{\text{deck},i}^{\text{total}}=\frac{\sigma_{\text{deck},i}}{E_{\text{deck}}}+\varepsilon_{\text{deck},i}. \end{array}$$

The element stresses result from the track and deck axial forces, from the connection forces of the ballast, and from any additional exterior longitudinal force, *F*_long_, as follows:

(2) $$\begin{array}{c} \sigma_{\text{rail},i}=\frac{N_{\text{rail},i}}{A_{\text{rail}}}=\frac{N_{\text{rail},i-1}-F_{\text{long},i}-F_{\text{ballast}}\left(u_{i}\right)}{A_{\text{rail}}}. \end{array}$$

The deck stress also depends on the restoring forces of piers and bearings, *F*_pier_, which can be determined from their stiffness by the longitudinal displacement of the corresponding node. Different stiffness for different vertical bearing loads can be considered:

(3) $$\begin{array}{c} \sigma_{\text{deck},i}=\frac{N_{\text{deck},i}}{A_{\text{deck}}}=\frac{N_{\text{deck},i-1}-F_{\text{pier},i}+F_{\text{ballast}}\left(u_{i}\right)}{A_{\text{deck}}}. \end{array}$$

The imposed strains are those resulting from temperature change, creep and shrinkage, or vertical deflection of the deck:

(4) $$\begin{array}{c} \varepsilon_{\text{rail},i}^{}=\alpha_{T,\text{rail}}\cdot \Delta T_{\text{rail}}+\varepsilon_{\text{vert}},\\ \varepsilon_{\text{deck},i}^{}=\alpha_{T,\text{deck}}\cdot \Delta T_{\text{deck}}+\varepsilon_{\text{vert}}+\varepsilon_{C+S}. \end{array}$$

Considering the relative displacement history from any previous load and the actual value of the relative displacement, it is possible to determine the actual connection force between these nodes. This force is taken as the basis for the analysis of the next, right-hand, element.

In that way, all connection forces and all node displacements of the complete bridge length can be calculated successively. The authors call this method the relative displacement method.

### 3.2. Solution Algorithm

The relative displacement of the first pair of nodes *i* may be arbitrary. Its correct value must be determined by an iterative optimisation algorithm, such that the boundary conditions of the bridge project are fulfilled. The precision of the correct value must be very high, especially in long viaducts (over 500 m), because small deviations will sum up to a large error. Only one solution will fulfil the boundary conditions.

Good boundary conditions are zero stress at rail or deck expansion joints, zero deck displacement at fixed bearings, or any particular stress value on the embankment on a sufficient distance from the bridge. In the optimisation algorithm, the relative displacement of the first pair of nodes *i* is varied until all of the boundary conditions are fulfilled. Each iteration requires the calculation of the complete bridge length.

In Algorithm 1, the outline of the calculation algorithm is shown for the example of a bridge with two rail expansion joints.

### 3.3. Definition of the Connector Behaviour

As described in Section 2.1, the mechanical behaviour of the rail-deck connection is rather complex. The usual finite element programs do not offer connector elements with such characteristics. It must be composed of a combination of various elements and subroutines or it might even be impossible to model.

The advantage of the proposed relative displacement method is that the connector behaviour can be defined directly as a mathematical function in the chosen programming language. This function can consider any parameters or results of the analysis.

**Figure 4:**
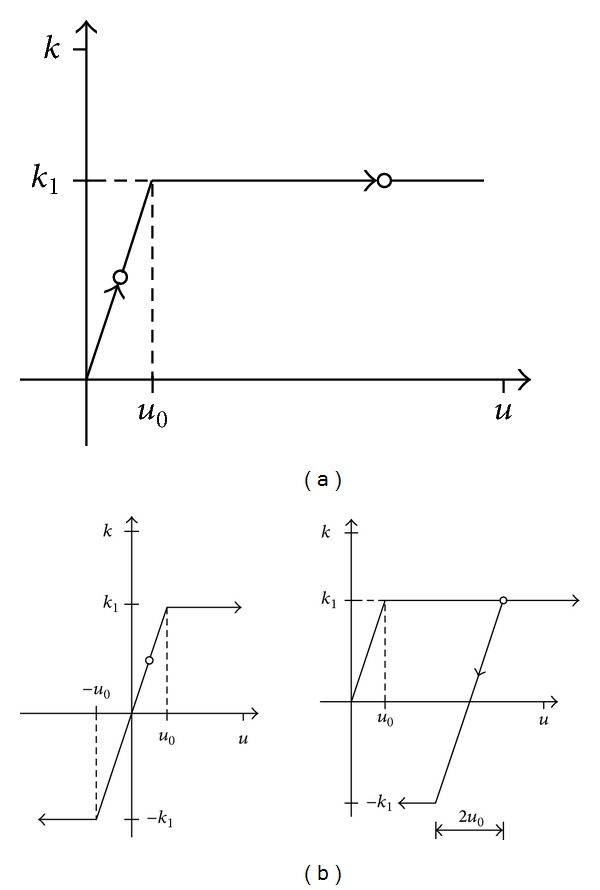
Rail-deck connection behaviour (a) for creep and shrinkage and (b) for subsequent temperature variation.

For example, for the analysis of creep and shrinkage and subsequent temperature variation, the six different connector behaviours shown in Figure 4 can be distinguished. At the end of the first step, the creep and shrinkage strain, two different states of the connector are possible: elastic or plastic behaviour. The subsequent temperature variation can produce a displacement in the same direction as the before step, or it can be contrariwise. If it is in the same direction, the connector behaviour will be the same as previous, and if it is contrariwise, it will be elastic but without recovering the possible previous plastic deformation. Furthermore, the final state of the connector can be elastic or plastic. This load-displacement behaviour can be described as follows:

(5) $$\begin{array}{c} F_{\text{ballast},C+S}=\{\begin{array}{cc} u_{C+S}\cdot \frac{k}{u_{0}}, & \left|u_{C+S}\right|<u_{0},\\ \mathrm{sgn}\left(u_{C+S}\right)\cdot k, & \left|u_{C+S}\right|\geq u_{0}, \end{array}\\ F_{\text{ballast},\Delta T}=\{\begin{array}{cc} \mathrm{sgn}\left(u_{C+S}\right)=\mathrm{sgn}\left(u_{\Delta T}\right) & \{\begin{array}{cc} \left(u_{C+S}+u_{\Delta T}\right)\cdot \frac{k}{u_{0}}, & \left|u_{C+S}+u_{\Delta T}\right|<u_{0},\\ \mathrm{sgn}\left(u_{C+S}\right)\cdot k, & \left|u_{C+S}+u_{\Delta T}\right|\geq u_{0}, \end{array}\\ \mathrm{sgn}\left(u_{C+S}\right)\neq \mathrm{sgn}\left(u_{\Delta T}\right) & \{\begin{array}{cc} \left|u_{C+S}\right|<u_{0}, & \{\begin{array}{cc} \left(u_{C+S}+u_{\Delta T}\right)\cdot \frac{k}{u_{0}}, & \left|u_{C+S}+u_{\Delta T}\right|<u_{0},\\ \mathrm{sgn}\left(u_{\Delta T}\right)\cdot k, & \left|u_{C+S}+u_{\Delta T}\right|\geq u_{0}, \end{array}\\ \left|u_{C+S}\right|\geq u_{0}, & \{\begin{array}{cc} \left(\mathrm{sgn}\left(u_{C+S}\right)\cdot u_{0}+u_{\Delta T}\right)\cdot \frac{k}{u_{0}}, & \left|u_{\Delta T}\right|<2\cdot u_{0},\\ \mathrm{sgn}\left(u_{\Delta T}\right)\cdot k, & \left|u_{\Delta T}\right|\geq 2\cdot u_{0}. \end{array} \end{array} \end{array} \end{array}$$

In this manner, it is possible to define any connector behaviour, even for the more complex cases when loaded and unloaded tracks have to be considered.

## 4. Application of the Proposed Method

**Figure 5:**
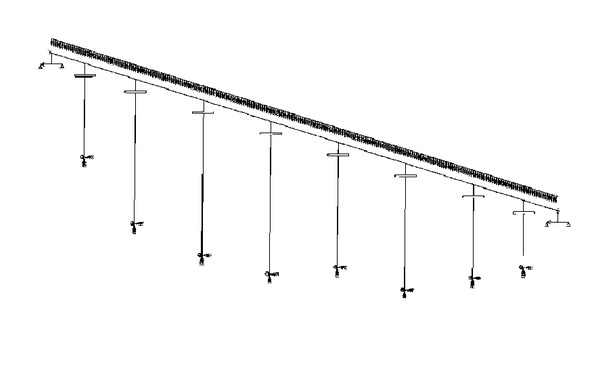
FEM bridge model used in ABAQUS.

To evaluate the validity of the proposed relative displacement method of the track-structure interaction, in the following it is applied to a real bridge example. The results are compared with those obtained from a conventional finite elements analysis performed in ABAQUS Standard software.  Figure 5 shows the FEM bridge model that was used. The bridge selected for the comparison is the Giles Viaduct of the AVE high speed railway track from Los Gallardos to Sorbas in Spain. It has a prestressed concrete box girder with a total length of 360 m divided into 8 spans. This bridge has one rail joint and one deck expansion joint at each abutment. The thermal centre is located in the centre of the bridge. The necessary analysis parameters are taken from the Spanish railway bridge design code [13].  Table 1 shows the most important of them.

The loads evaluated are, in the first step, the deck deformation due to creep and shrinkage at infinite time. In the second step, based on the equilibrium state of the first load case, the variation of the rail temperature is applied, in this case a temperature increase of 20 K.

**Figure 6:**
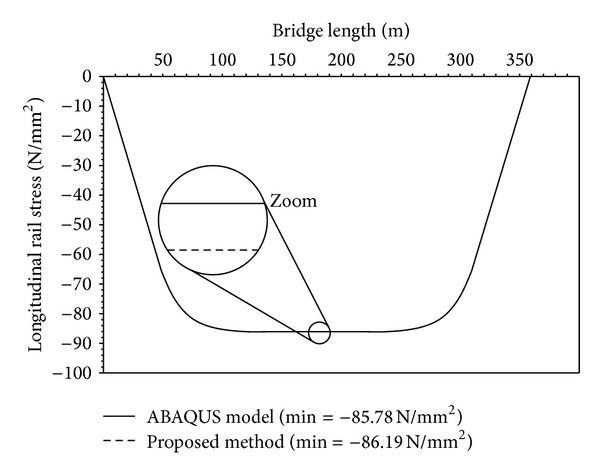
Rail stress due to creep and shrinkage deformation.

Figure 6 shows the results for the rail stress of the first load case for both the ABAQUS and the relative displacement analysis. Both graphs are plotted in the same diagram but cannot be distinguished because they are virtually the same. The minimum rail stress value of −85.78 N/mm^2^ is identical for both analysis methods.

**Figure 7:**
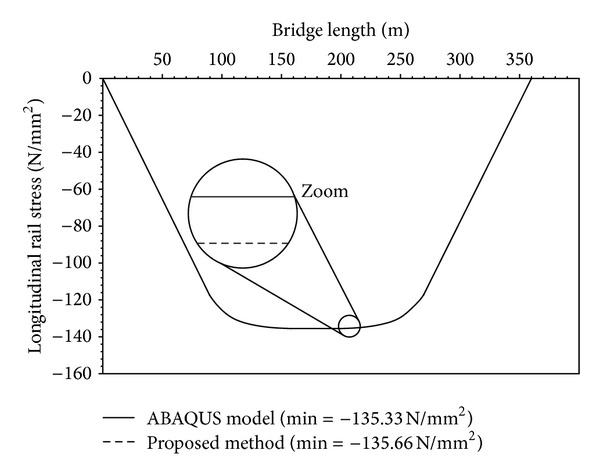
Rail stress due to creep, shrinkage, and temperature deformation.

The rail stress for the second load case, a rail temperature increment of +20 K, is obtained by applying a subsequent rail deformation to the analysis model equilibrium state after creep and shrinkage.  Figure 7 shows the resulting rail stress, both for the ABAQUS model and for the relative displacement method. As before, the corresponding graphs cannot be distinguished in the diagram because they are virtually the same. The minimum rail stress values, 135.33 N/mm^2^ from ABAQUS and 135.66 N/mm^2^ from the proposed method, are identical in practical terms (0.3% deviation).

In this example and as experienced in 14 other railway viaducts with lengths from 123 m to 2,525.5 m, the results of the conventional FEM analysis and of the relative displacement method are of equal quality. The CPU time was instantaneous for both methods, while the model preparation time before analysis for an experienced user was about half a day for the ABAQUS model and less than half an hour for the relative displacement method. This comparison takes into account that a general model of the bridge is already available in ABAQUS from the bridge design process.

## 5. Summary and Conclusions

The track-structure interaction in railway bridges is commonly calculated with finite element analysis software. In the case of ballasted tracks, the connection between track and structure has a nonlinear, plastic, and irreversible mechanical behaviour that depends moreover on the vertical load applied to the viaduct. Most of the commercial software is not prepared for the implementation of such elements.

To find a less complex method, the problem was reduced to single DOF finite elements, and an iterative optimisation algorithm was proposed in place of the solution of the equilibrium equation system by means of the stiffness matrix. This method can be programmed in any language or even in spreadsheet applications. The definition of any mechanical behaviour of the track-structure connector is easily possible.

In the proposed method, an initial relative track-structure displacement is assumed at one node, and subsequently, all node forces and displacements of the deck and the track are calculated. Exterior forces acting on the track or on the structure, such as traction and braking force or bearing restoring force, can be taken into account. Furthermore, all imposed deck or track deformations, such as creep, shrinkage, or thermal expansion, are implemented.

The correct value of the initial relative track-structure displacement is determined by an iterative optimisation algorithm. It is obtained when the calculated deformation state of the model fulfils all the boundary conditions of the viaduct, for example, zero stress at expansion joints.

The comparison of this proposed relative displacement method with an ABAQUS analysis model shows that both results are of the same quality and that their rail stress values are virtually identical. In terms of time consumption, the relative displacement method is very advantageous because the preparation time before analysis is less than half an hour, while it is half a day for the ABAQUS analysis model.

The proposed method has certain limitation because the deformation of the whole bridge is calculated starting from one node. A very high precision of the deformation values is necessary; otherwise, small deviations will sum up to a large error. The precision of EXCEL spreadsheets is sufficient for up to 500 m long viaducts; with FORTRAN a 2,525.5 m long bridge was calculated successfully.

## Figures and Tables

**Algorithm 1:**
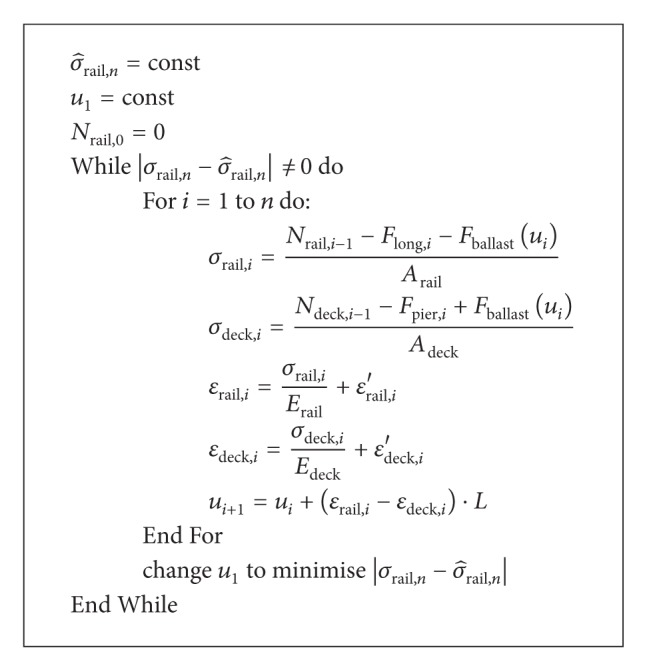


**Table 1:** Parameters of Giles Viaduct, Spain.

Bridge length	24 m + 36 m + 5 × 48 m + 36 m + 24 m = 360 m
Track number	2
Deck cross-section	10.198 m^2^
Rail cross-section	4 × 7,678 mm^2^ = 30,712 mm^2^
Plastic shear resistance *k *	20 kN/m
Relative displacement elastic limit *u*_0_	2 mm
Creep and shrinkage strain	−4.56*E* − 2‰
Rail temperature increment Δ*T*	+20 K
Coefficient of thermal expansion	
Deck	1.00*E* − 5 K^−1^
Rail	1.20*E* − 5 K^−1^

## Notations

*n*: Total number of nodes
*k*: Plastic shear resistance of the track
*u*: Relative track-structure displacement
*u*_0_: Elastic limit of the relative track-structure displacement
*A*: Cross-section area
*E*: Young's modulus
*F*: Longitudinal force
*L*: Element length
*N*: Axial force
*α*_*T*_: Coefficient of thermal expansion
*x*: Strain
*σ*: Stress
$\hat{\sigma }$: Boundary condition stress
Δ*T*: Temperature variation.
